# Development and in vitro Profiling of Dual FXR/LTA4H Modulators

**DOI:** 10.1002/cmdc.202100118

**Published:** 2021-05-24

**Authors:** Simone Schierle, Steffen Brunst, Moritz Helmstädter, Roland Ebert, Jan S. Kramer, Dieter Steinhilber, Ewgenij Proschak, Daniel Merk

**Affiliations:** ^1^ Institute of Pharmaceutical Chemistry Goethe University Frankfurt Max-von-Laue-Str. 9 60438 Frankfurt Germany

**Keywords:** farnesoid X receptor, leukotriene A4 hydrolase, polypharmacology, non-alcoholic steatohepatitis, non-alcoholic fatty liver disease

## Abstract

Designed polypharmacology presents as an attractive strategy to increase therapeutic efficacy in multi‐factorial diseases by a directed modulation of multiple involved targets with a single molecule. Such an approach appears particularly suitable in non‐alcoholic steatohepatitis (NASH) which involves hepatic steatosis, inflammation and fibrosis as pathological hallmarks. Among various potential pharmacodynamic mechanisms, activation of the farnesoid X receptor (FXRa) and inhibition of leukotriene A4 hydrolase (LTA4Hi) hold promise to counteract NASH according to preclinical and clinical observations. We have developed dual FXR/LTA4H modulators as pharmacological tools, enabling evaluation of this polypharmacology concept to treat NASH and related pathologies. The optimized FXRa/LTA4Hi exhibits well‐balanced dual activity on the intended targets with sub‐micromolar potency and is highly selective over related nuclear receptors and enzymes rendering it suitable as tool to probe synergies of dual FXR/LTA4H targeting.

## Graphical abstract

**Polypharmacology by design**: FXR agonists (FXRa) and LTA4H inhibitors (LTA4Hi) hold therapeutic potential in non‐alcoholic steatohepatitis (NASH). We report the design and characterization of a potent FXRa/LTA4Hi with balanced dual activity and high selectivity over related transcription factors and enzymes. It may serve as a pharmacological tool to evaluate potential synergies of dual FXR/LTA4H modulation in NASH.

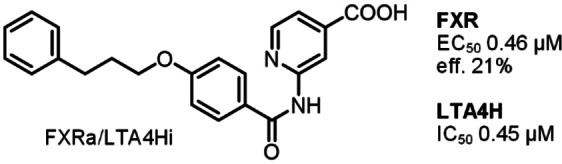

## Introduction

Designed polypharmacology^[1]^ is increasingly recognized as a promising strategy to obtain safer and more efficacious drugs. It particularly presents as a reasonable approach to address multifactorial diseases where a single mode of action may be insufficient for therapeutic success. Non‐alcoholic fatty liver disease (NAFLD) is such highly multifactorial pathology[^2^, ^3^] for which designed polypharmacology is probed as potentially superior therapeutic approach. It is the most prevalent chronic liver disorder in the world affecting approx. 25 % of the adult population.[^4^, ^5^] NAFLD and its progressed form non‐alcoholic steatohepatitis (NASH) are considered as hepatic manifestation of the metabolic syndrome and are closely associated with various metabolic disorders such as type 2 diabetes.^[5]^ The disease complex involves various pathological factors including metabolic imbalance, hepatic fat accumulation, inflammation, oxidative cell damage, and fibrosis. Designed polypharmacology addressing complementary pathomechanisms may, therefore, offer access to synergistic efficacy in this complex disease. Following this strategy, we have previously developed dual modulators to target, for example, farnesoid X receptor (FXR) and soluble epoxide hydrolase (sEH),[^6^, ^7^, ^8^] or FXR and peroxisome proliferator‐activated receptors (PPARs).[^9^, ^10^] Strong therapeutic efficacy of such multi‐target agents in preclinical NASH models[^10^, ^11^] corroborates the potential of designed polypharmacology in this indication. We further hypothesize that a combination of partial FXR activation^[12]^ and inhibition of leukotriene A4 hydrolase (LTA4H) can efficiently counteract NAFLD/NASH.

FXR is a ligand‐activated transcription factor and acts as cellular sensor for bile acids with chenodeoxycholic acid (CDCA) as the most active endogenous ligand.[^13^, ^14^] It is involved in the maintenance of bile acid, lipid and glucose homeostasis, and is considered as key liver protective transcriptional regulator.^[15]^ FXR activation has been broadly studied in clinical trials as strategy to treat metabolic liver diseases including NAFLD/NASH.[^16^, ^17^] The semisynthetic FXR agonist obeticholic acid (OCA, **1**)^[18]^ has validated FXR as a therapeutic target and is leading the NASH pipeline which also contains several synthetic FXR agonists derived from GW4064 (**2**)^[19]^ and other chemotypes.^[20]^

Leukotriene A4 hydrolase (LTA4H) converts the arachidonic acid derived leukotriene A4 (LTA4) into the pro‐inflammatory and chemotactic mediator leukotriene B4 (LTB4). Leukotrienes contribute to hepatic inflammation in NAFDL^[21]^ suggesting therapeutic potential of LTA4H inhibition to counteract steatohepatitis. Moreover, LTA4H inhibition shunts LTA4 conversion to the specialized pro‐resolving mediator lipoxin A4 (LXA4)^[22]^ which was found to promote resolution of hepatic inflammation and diminish fibrotic changes in the setting of NASH.^[23]^ Additionally, LXA4 and LTB4 are linked to cholesterol biosynthesis^[24]^ indicating potential synergy with FXR ligands since FXR is the master regulator of bile acid biosynthesis from cholesterol.^[14]^

Following these considerations, we aimed to develop a tool to probe potential additive or synergistic efficacy of dual FXR/LTA4H modulation in NASH and other metabolic diseases. We have screened for dual modulators of FXR and LTA4H and systematically elucidated the structure‐activity relationship (SAR) of **3** as a promising dual modulator chemotype to obtain a potent and balanced FXR activator (FXRa) and LTA4H inhibitor (LTA4Hi). Here we report the development, SAR and preliminary profiling of this new designed multi‐target agent.

## Results and Discussion

**Scheme 1:**
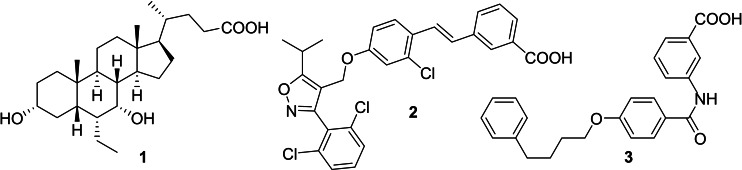
Chemical structures of FXR agonists obeticholic acid (**1**) and GW4064 (**2**), and the FXRa/LTA4Hi lead **3**.

**Scheme 2:**
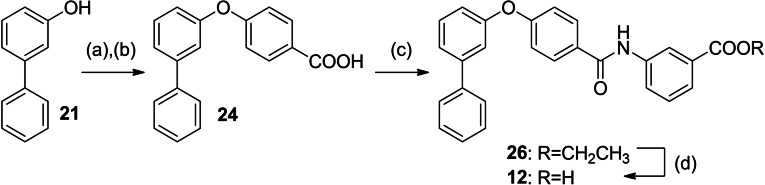
Synthesis of **12**: (a) (4‐(Ethoxycarbonyl)phenyl)boronic acid (**22**), Cu(OAc)_2_, pyridine, DMF, 90 °C, 12 h, 66 %; (b) LiOH, THF, EtOH, H_2_O, 50 °C, 12 h, 95 %; (c) ethyl 3‐aminobenzoate (**25**), EDC⋅HCl, 4‐DMAP, CHCl_3_, reflux, 6 h, 36 %; (d) LiOH, THF, EtOH, H_2_O, 50 °C, 12 h, 74 %.

**Scheme 3:**
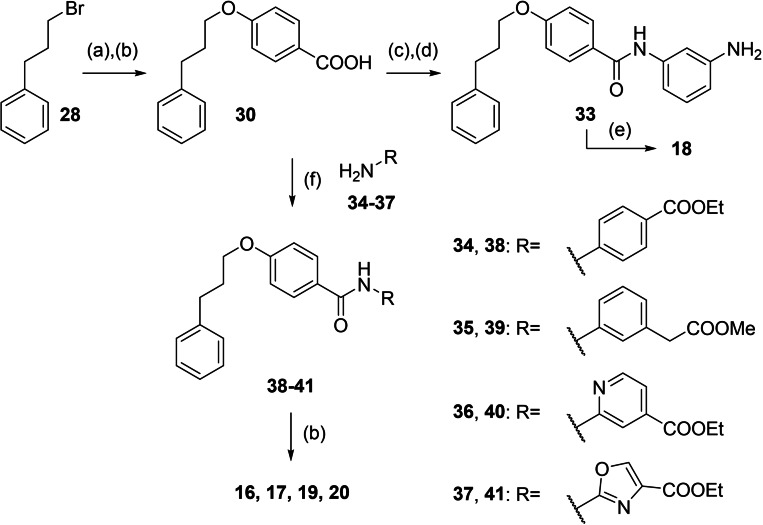
Synthesis of **16**–**20**: (a) Methyl 4‐hydroxybenzoate (**27**), Cs_2_CO_3_, DMF, 90 °C, 10 h, 80 %; (b) LiOH, THF, EtOH, H_2_O, 50 °C, 12 h, 27–86 %; (c) 3‐nitroaniline (**31**), EDC⋅HCl, 4‐DMAP, CHCl_3_, reflux, 6 h, 80 %; (d) Pd(C), H_2_, EtOAc, rt, 12 h, 88 %; (e) methanesulfonyl chloride, TEA, THF, rt °C, 6 h, 27–76 %; (f) EDC⋅HCl, 4‐DMAP, CHCl_3_, reflux, 8 h, 18–80 %.

In screening selected compounds of our in‐house collection of FXR ligands for LTA4H inhibition, **3** ^[25]^ (Scheme 1, Table 1) evolved as an appealing FXRa/LTA4Hi lead compound with high partial agonist potency on FXR (EC_50_ 0.026 μM, 34 % efficacy) and intermediate LTA4H inhibitory activity (IC_50_ 0.54 μM). To obtain a more balanced FXRa/LTA4Hi as tool, we systematically probed the structure‐activity relationship of the dual modulator chemotype with analogues **4**–**20**. The synthesis of **3**, **5**–**11** and **13**–**15** has been reported previously.^[25]^ **12** and **16**–**20** were prepared according to Schemes 2 and 3. For the synthesis of the rigidified dual modulator **12** (Scheme 2), 3‐hydroxybiphenyl (**21**) was treated with (4‐(ethoxycarbonyl)phenyl)boronic acid (**22**) in presence of Cu(OAc)_2_ to obtain **23** followed by ester hydrolysis to **24**. Subsequent amide coupling with ethyl 3‐aminobenzoate (**25**) afforded **26** and ester hydrolysis generated **12**.

**Table 1:** In vitro biological activity of **3**–**12**.

ID	Structure	EC_50_(FXR)^[a]^ (efficacy)	IC_50_(LTA4H)^[b]^ (max. inhib.)
**3**	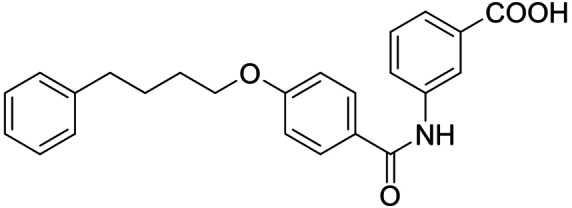	0.026±0.001 μM (34±1 %)	0.54±0.07 μM (97.2±0.4 %)
**4**	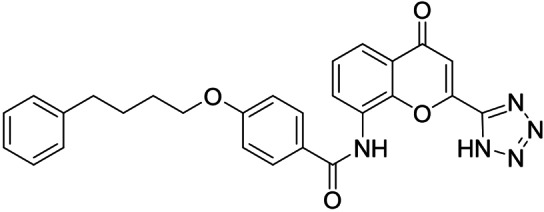	15±3 μM (27±1 %)	1.6±0.2 μM (82.6±0.8 %)
**5**	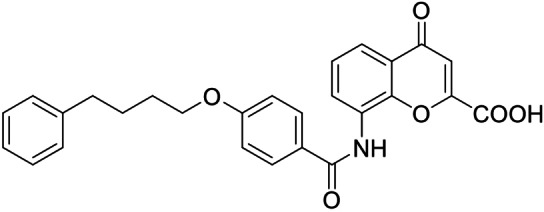	inactive (50 μM)	15±2 μM (80±5 %)
**6**	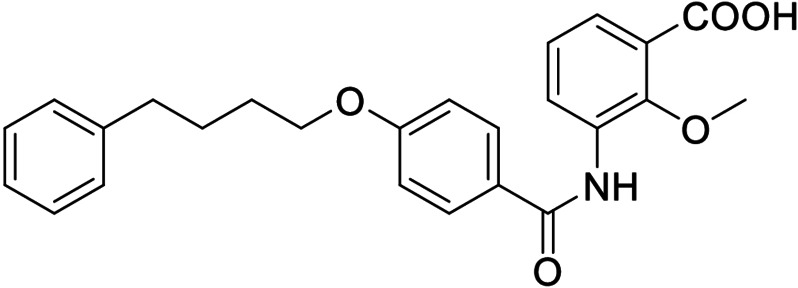	0.19±0.02 μM (26±1 %)	5.5±0.5 μM (85±2 %)
**7**	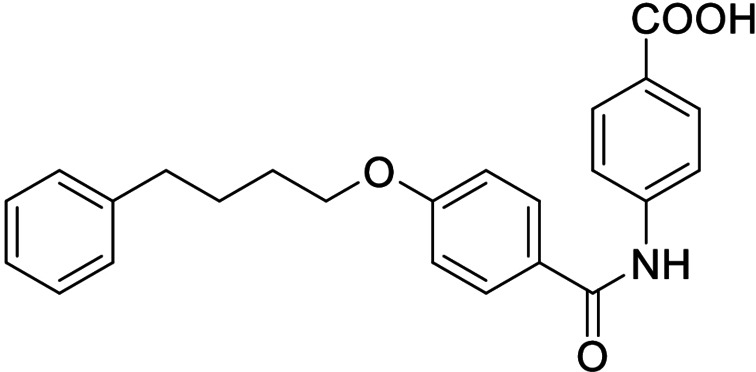	0.014±0.001 μM (29±1 %)	0.37±0.02 μM (97.8±0.1 %)
**8**	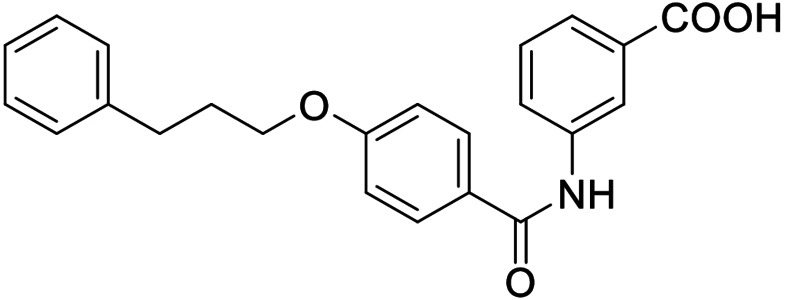	0.029±0.006 μM (22±1 %)	0.14±0.01 μM (98.8±0.1 %)
**9**	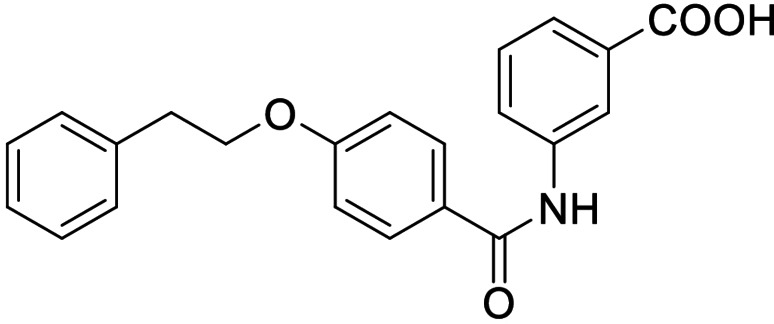	0.15±0.02 μM (56±1 %)	0.61±0.06 μM (96.0±0.2 %)
**10**	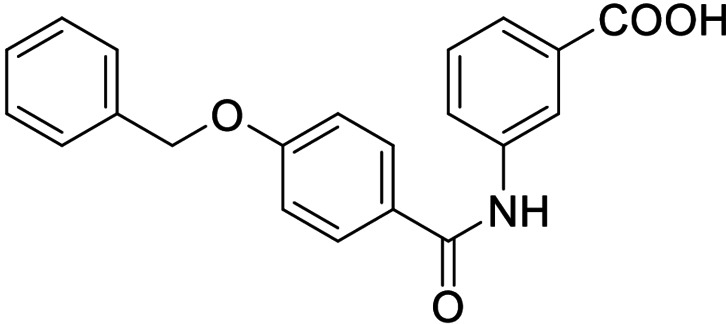	0.009±0.001 μM (20±1 %)	1.1±0.1 μM (94.9±0.2 %)
**11**	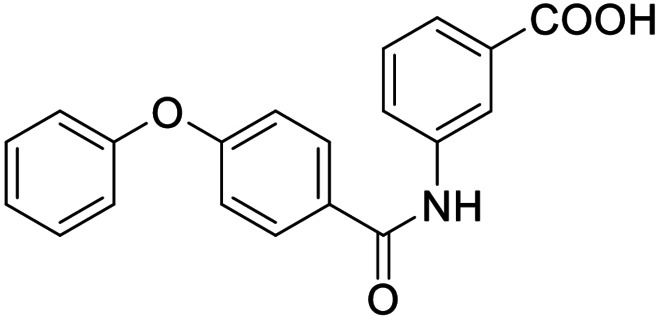	0.0010±0.0003 μM (35±1 %)	0.55±0.06 μM (98.2±0.1 %)
**12**	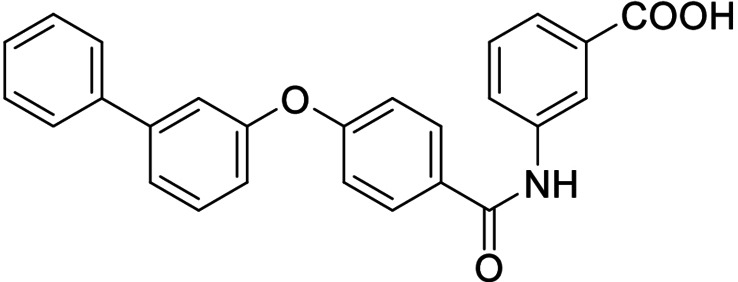	0.3±0.1 μM (14±1 %)	5.2±0.6 μM (81.1±0.6 %)

[a] FXR modulation was determined in a full‐length FXR reporter gene assay based on the human FXR response element from the BSEP promoter. Efficacy refers to maximum FXR activation relative to the activity of 3 μM GW4064 which was defined as 100 % activation. The activity of **3**–**11** on FXR has been previously reported^[25]^. Data are the mean±S.E.M., n≥3. [b] LTA4H inhibition was determined on recombinant protein using L‐arginine‐7‐amino‐4‐methylcoumarine as fluorogenic substrate. Maximum inhibition (max. inhib.) refers to LTA4H inhibition at the highest tested concentration. Data are the mean±S.E.M., n=3.

Synthesis of the sulfonamide analogue **18** (Scheme 3) commenced with a Williamson ether synthesis using methyl 4‐hydroxybenzoate (**27**) and 3‐bromopropylbenzene (**28**) to obtain **29** followed by ester hydrolysis to **30**. Amide coupling with 3‐nitroaniline (**31**) resulted in **32** and subsequent reduction yielded aniline **33**, which was suitable for sulfonamide synthesis to **18**. Dual modulators **16**, **17**, **19** and **20** were also prepared from precursor **30** by amide coupling with anilines **34**–**37** to intermediates **38**–**41** and subsequent ester hydrolysis.

FXR modulation by **3–20** was characterized in a cellular (HeLa, transiently transfected) full‐length FXR reporter gene assay employing the human FXR response element from the promoter region of bile salt export protein (BSEP) to govern firefly luciferase expression as reporter gene.[^26^, ^27^] FXR and its natural heterodimer partner retinoid X receptor (RXR) were constitutively over‐expressed and a constitutively expressed renilla luciferase was present as internal control for transfection efficiency and cellular health. The reference FXR agonist GW4064 (**2**, 3 μM defined as 100 % efficacy) served as positive control, DMSO (0.1 %) treated cells as negative control. LTA4H inhibition by **3**–**20** was determined in a fluorogenic assay on recombinant LTA4H protein with L‐arginine‐7‐amino‐4‐methylcoumarine as substrate which is cleaved by the enzyme to L‐arginine and fluorescent 7‐amino‐4‐methylcoumarine. Bestatine served as reference LTA4H inhibitor.

The FXR ligand **3** had been originally derived from pranlukast (**4**)^[25]^ and is characterized as a partial agonist with intermediate FXR activation efficacy which is an attractive characteristic[^12^, ^28^] considering adverse effects such as a disturbed cholesterol homeostasis[^16^, ^17^] that have been observed with strong FXR activators. The structural similarity of the lead **3** to pranlukast (**4**) prompted us to probe the LTA4H inhibitory potency of the drug compound **4** and close analogues **5** and **6** from the previous SAR study on FXR, too (Table 1). Pranlukast (**4**) revealed considerable LTA4H inhibitory activity, as well, while the chromenonecarboxylate **5** and the ortho‐methoxybenzoate analogue **6** mimicking the chromenone were significantly less active rendering the unsubstituted benzoic acid **3** as preferred lead. Also shifting the benzoic acid regiochemistry from meta‐ (**3**) to para‐substitution (**7**) was not superior.

Consequently, we based further dual SAR elucidation on lead compound **3**. In the lipophilic ether substituent of **3**, stepwise chain shortening from phenylbutyloxy (**3**) over phenylpropyloxy (**8**), phenylethoxy (**9**) and benzyloxy (**10**) to phenoxy (**11**) had promoted partial FXR agonist potency to single digit nanomolar EC_50_ values.^[25]^ Regarding LTA4H inhibition, an optimal chain length was achieved in phenylpropyloxy analogue **8** with 0.14 μM IC_50_ value while shorter chains were less active. Additionally, due to its exceptional potency on FXR, phenoxy derivative **11** also appeared favorable for further evaluation. A rigid combination of **8** and **11**, obtained by introduction of a 3‐biphenylether in **12** decreased FXR activation efficiency to low 14 % activation and caused a pronounced loss in LTA4H inhibitory potency.

As no balanced dual modulator was discovered with the previously reported phenoxy‐substituted series **13–15** (Table 2), we studied further head group modifications with the phenylpropyloxy motif of **8** (Table 3). The 4‐aminobenzoic acid regiochemistry (**7**) had been slightly preferred on both targets in the early SAR elucidation. Hence, we combined it with the phenylpropyloxy substituent in **16** which was a potent dual modulator but not superior to **8**. We then probed the effect of chain elongation from 3‐aminobenzoic acid (**8**) to 3‐aminophenylacetic acid (**17**) which was not favored by LTA4H, however, and caused a marked decrease in FXR activation activity. A methylsulfonamide motif (**18**) as carboxylic acid bioisostere, which had enabled the design of a highly potent dual FXR/sEH modulator,[^6^, ^11^] did not alter potency on LTA4H but diminished FXR agonism, as well. In the phenoxy series, a 2‐aminoisonicotinic acid (**15**) head group was favored by both targets especially in terms of FXR activation efficacy. Thus, we fused this modification with the LTA4H favored phenylpropyloxy substituent in **19** which resulted in very well balanced dual modulator activity. In an attempt to further enhance polarity, we replaced the pyridine motif of **19** with an oxazole in **20** but this modification markedly diminished activity on FXR.

**Table 2:** In vitro biological activity of **13**–**15. 11** for comparison.

ID	Structure	EC_50_(FXR)^[a]^ (efficacy)	IC_50_(LTA4H)^[b]^ (max. inhib.)
**11**	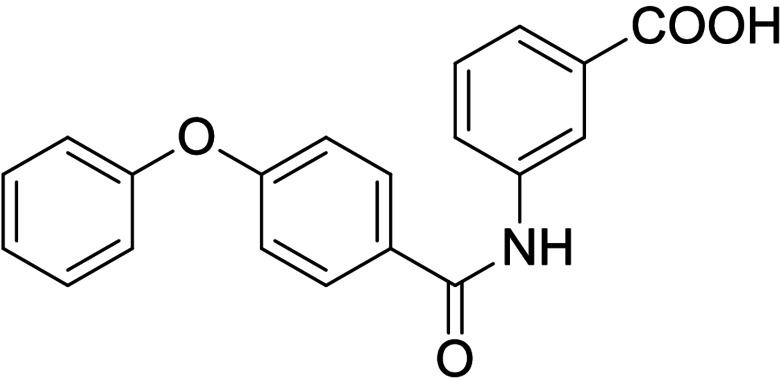	0.0010±0.0003 μM (35±1 %)	0.55±0.06 μM (98.2±0.1 %)
**13**	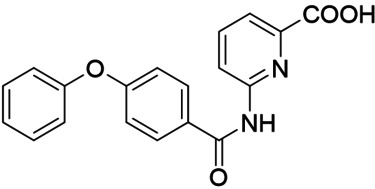	*antagonist*	0.28±0.02 μM (90.8±0.3 %)
**14**	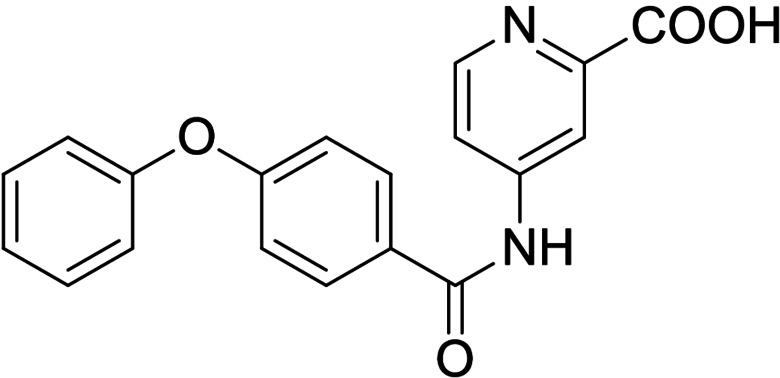	0.017±0.006 μM (21±1 %)	0.95±0.01 μM (97.9±0.1 %)
**15**	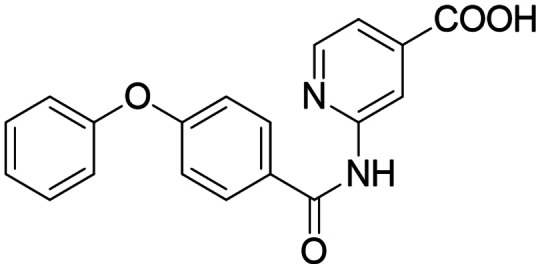	0.07±0.02 μM (56±1 %)	2.1±0.1 μM (89.6±0.6 %)

[a] FXR modulation was determined in a full‐length FXR reporter gene assay based on the human FXR response element from the BSEP promoter. Efficacy refers to maximum FXR activation relative to the activity of 3 μM GW4064 which was defined as 100 % activation. The activity of **13–15** on FXR has been previously reported^[25]^. Data are the mean±S.E.M., n≥3. [b] LTA4H inhibition was determined on recombinant protein using L‐arginine‐7‐amino‐4‐methylcoumarine as fluorogenic substrate. Maximum inhibition (max. inhib.) refers to LTA4H inhibition at the highest tested concentration. Data are the mean±S.E.M., n=3.

**Table 3:** In vitro biological activity of **16**–**20. 8** for comparison.

ID	Structure	EC_50_(FXR)^[a]^ (efficacy)	IC_50_(LTA4H)^[b]^ (max. inhib.)
**8**	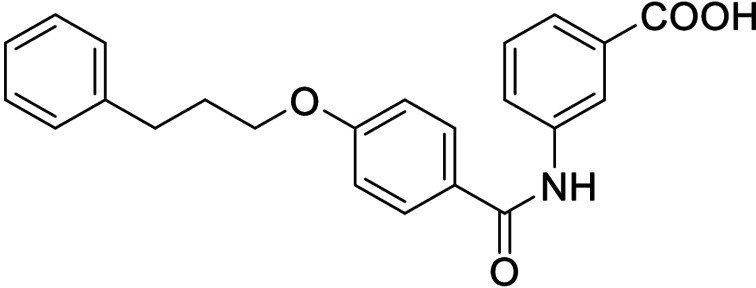	0.029±0.006 μM (22±1 %)	0.14±0.01 μM (98.8±0.1 %)
**16**	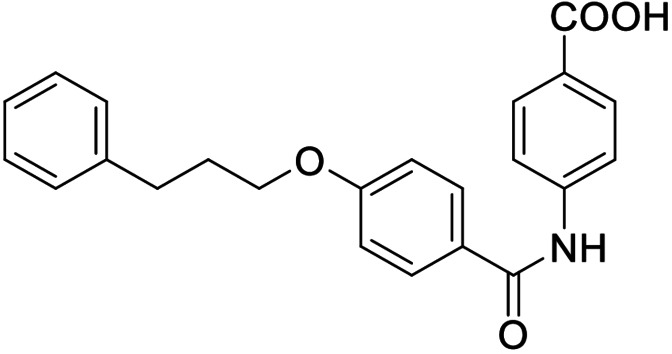	>30 μM	0.20±0.02 μM (98.8±0.1 %)
**17**	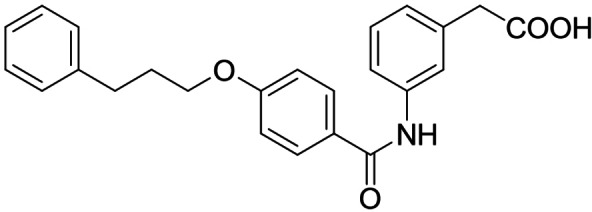	>30 μM	1.9±0.1 μM (97.0±0.2 %)
**18**	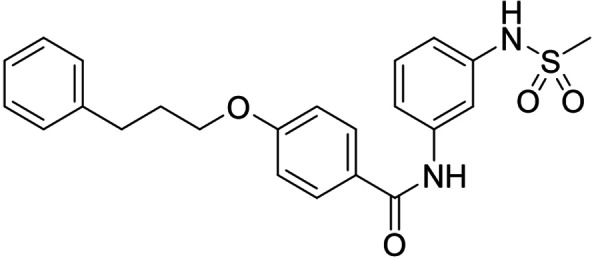	1.6±0.4 μM (35±2 %)	0.13±0.01 μM (89.5±0.5 %)
**19**	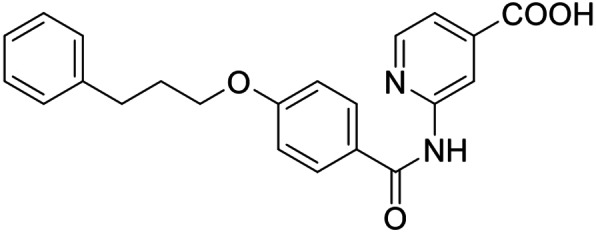	0.46±0.08 μM (21±1 %)	0.45±0.02 μM (98.9±0.1 %)
**20**	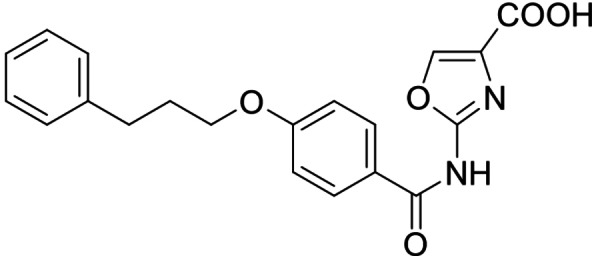	11±2 μM (30±1 %)	0,16±0,02 μM (99.7±0.1 %)

[a] FXR modulation has been determined in a full‐length FXR reporter gene assay based on the human FXR response element from the BSEP promoter. Efficacy refers to maximum FXR activation relative to the activity of 3 μM GW4064 which was defined as 100 % activation. Data are the mean±S.E.M., n≥3. [b] LTA4H inhibition was determined on recombinant protein using L‐arginine‐7‐amino‐4‐methylcoumarine as fluorogenic substrate. Maximum inhibition (max. inhib.) refers to LTA4H inhibition at the highest tested concentration. Data are the mean±S.E.M., n=3.

From our dual SAR study, **8** and **19** evolved as attractive early tools to study potential synergies arising from dual FXR activation and LTA4H inhibition. With an EC_50_ value of 0.46 μM for partial FXR agonism and an IC_50_ value of 0.45 μM on LTA4H, **19** exhibits well‐balanced sub‐micromolar activity on the two desired targets whereas **8** is more active (FXR: EC_50_ 0.029 μM; LTA4H: IC_50_ 0.14 μM) but less balanced. Both compounds appeared suitable for further profiling prompting us to characterize their properties, biological activities and selectivity in vitro.

**Figure 1:**
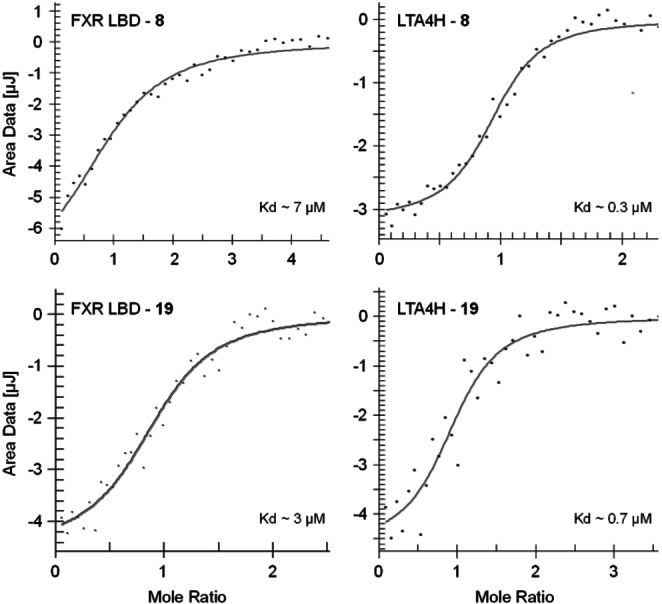
Isothermal titration calorimetry (ITC) confirmed binding of **8** and **19** to the recombinant human FXR LBD^[29]^ and to recombinant human LTA4H.^[30]^ 1:1 binding was assumed.

**Figure 2:**
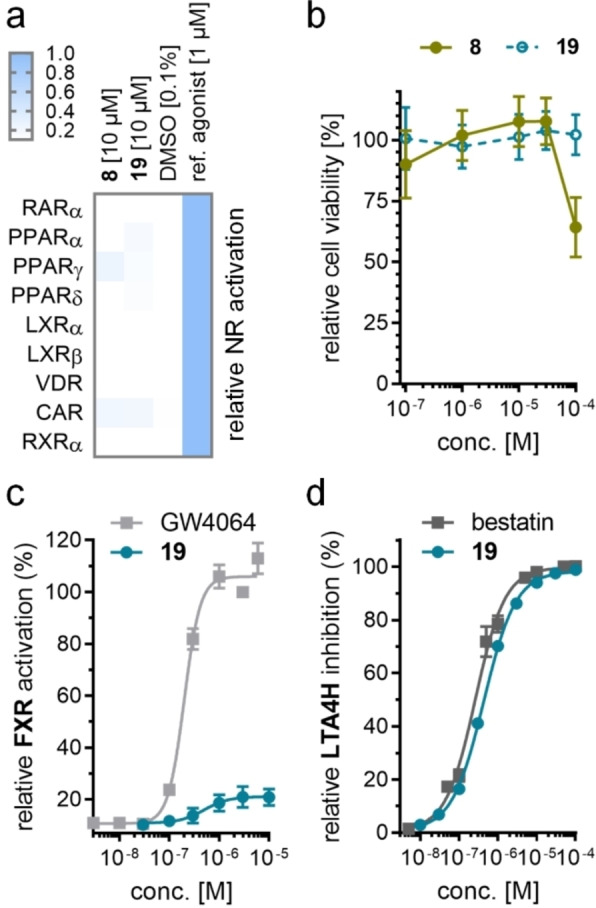
(a) Selectivity profiles of **8** and **19** over related nuclear receptors. Heatmap shows mean relative activation, n=2. (b) Activity of **8** and **19** in a WST‐1 cell viability assay in HepG2 cells. Data are mean±S.E.M., n=4. (c) Dose‐response of partial FXR agonist **19** on full‐length human FXR in a reporter gene assay based on the FXR response element from the promoter region of BSEP. Reference FXR agonist GW4064 (**2**) for comparison. Data are mean±S.E.M. relative FXR activation (%), n≥3. (d) Dose‐response of LTA4H inhibitor **19** on recombinant human LTA4H with L‐arginine‐7‐amino‐4‐methylcoumarine as fluorogenic substrate. Reference LTA4H inhibitor bestatin for comparison. Data are mean±S.E.M. relative LTA4H inhibition (%), n=3.

Isothermal titration calorimetry (ITC) confirmed binding of **8** and **19** to the recombinant proteins of both FXR LBD and LTA4H (Figure 1). Selectivity profiling on related targets within the nuclear receptor family (retinoic acid receptor, RAR; peroxisome proliferator‐activated receptors, PPAR; liver X receptors, LXR; vitamin D receptor, VDR; constitutive androstane receptor, CAR; retinoid X receptor; RXR; Figure 2a) demonstrated favorable selectivity of **8** and **19** despite weak PPARγ activation by **8**. Both compounds also did not inhibit soluble epoxide hydrolase (sEH), an enzyme of the arachidonic acid cascade which converts epoxyeicosatrienoic acids to the corresponding diols, at relevant concentrations (Table 4). This favorable selectivity profile is an important feature for FXRa/LTA4Hi tools to be used in experimental NASH treatment since inhibition of sEH has shown therapeutic efficacy in several NASH models,[^11^, ^32^, ^33^] too, affecting the outcome of pharmacological studies conducted with FXRa/LTA4Hi. Moreover, **19** caused no cytotoxic effects in a WST‐1 assay in HepG2 cells up to 100 μM concentration while **8** exhibited a slight anti‐proliferative activity at high concentration (Figure 2b).

**Table 4:** Selectivity of **8** and **19** over related targets within the arachidonic acid cascade.

	**8**	**19**
sEH inhibition^[a]^	6±1 % inhibition at 100 μM	IC_50_=68±2 μM

[a] sEH inhibition was determined on recombinant human sEH protein using (3‐phenyloxiranyl)acetic acid cyano‐(6‐methoxynaphthalen‐2‐yl)methyl ester (PHOME) as fluorogenic substrate^[31]^. Data are the mean±S.E.M., n=3.

Overall, the balanced activity on FXR and LTA4H (Figure 2c and d) combined with its high selectivity over related targets and the absence of cytotoxic activity presents **19** as most favorable dual FXRa/LTA4Hi for further investigation.

Retrospective molecular docking analysis of **19** as FXRa/LTA4Hi rationalized the SAR of the dual ligands. The predicted binding mode of **19** in the LTA4H active site (PDB ID 3FHE,^[34]^ Supporting Information Figure 1a) suggested that the carboxylate moiety undergoes metal interaction with the Zn^2+^ ion in the catalytic center. Despite not being crucial for LTA4H inhibition, this metal interactions contributes to binding affinity which agrees with the fact that almost all compounds of the chemotype displayed LTA4H inhibitory activity. The alkoxy motif of **19** points towards the hydrophilic tunnel. We have previously shown that the interaction with this hydrophobic tunnel plays a critical role in ligand binding to LTA4H.^[30]^ While the lipophilic tunnel can accept various alkoxy and phenoxy substituents, rigid moieties as present, for example, in **12** are not favored. In addition to the Zn^2+^‐interaction and occupation of the lipophilic tunnel, the amide carbonyl oxygen of **19** engages a directed H‐bond towards Gln134, while the NH is oriented towards the electron‐rich Tyr378. Molecular docking of **19** to the FXR ligand binding site (PDB ID 4QE8,^[12]^ Supporting Information Figure 1b) suggested a directed H‐bond between the Lys339 side chain and the amide carbonyl oxygen as well as the canonical ionic contact of the carboxylate group with Arg331 as major polar interactions. These observations provide an explanation why compounds with altered distance between these two key features such as, for example, **5**, **16**, and **17** substantially loose FXR activity. As observed for LTA4H, the alkoxy moiety of **19** occupies a hydrophobic tunnel which seems to tolerate diverse lipophilic groups.

## Conclusion

Multi‐factorial diseases involving multiple patho‐mechanisms and dysregulated signaling systems often require the sum of several pharmacodynamic mechanisms for efficacious therapeutic intervention. While this can be achieved by drug combinations, designed polypharmacology^[1]^ has well‐described advantages and evolves as potentially superior approach to treat multi‐factorial chronic diseases. One such disease is NASH which arises from metabolic imbalance, inflammation and fibrotic transformation in liver, and hence demands therapeutic intervention against several factors. Previous studies have shown that designed multiple ligands of FXR and sEH,[^6^, ^7^, ^8^] PPARα and PPARδ^[35]^ as well as FXR and PPARδ[^9^, ^10^] hold potential to achieve superior efficacy in experimental NASH treatment. Here we report a novel designed multiple ligand that concomitantly activates FXR and inhibits LTA4H. Strong evidence ascribes both molecular targets potential in NASH treatment wherein FXR has beneficial effects on hepatic metabolism and lipid homeostasis, and LTA4H is involved in hepatic inflammation and inflammation resolution. The FXRa/LTA4Hi **19** provides well balanced and potent dual activity combined with low toxicity and pronounced selectivity for its intended targets. **19** appears suitable as a tool to study the therapeutic potential of dual FXR/LTA4H modulation in NASH in early preclinical models warranting further development.

## Experimental Section

### Chemistry

**General**. All chemicals and solvents were of reagent grade and used without further purification unless otherwise specified. All reactions were conducted in oven‐dried glassware under argon atmosphere and in absolute solvents. NMR spectra were recorded on a Bruker AV 500, Bruker AV 400 or a Bruker am250xp spectrometer (Bruker Corporation, Billerica, MA, USA). Chemical shifts (δ) are reported in ppm relative to tetramethylsilane (TMS) as reference. Multiplicity is reported: s, singlet; d, doublet; dd, doublet of doublets; ddd, doublet of doublet of doublets; t, triplet; m, multiplet. Approximate coupling constants (*J*) are shown in hertz (Hz). Mass spectra were obtained on a VG Platform II (Thermo Fischer Scientific, Inc., Waltham, MA, USA) using electrospray ionization (ESI). High resolution mass spectra were recorded on a MALDI LTQ ORBITRAP XL instrument (Thermo Fisher Scientific). Compound purity was analyzed on a Waters 600 Controller HPLC (Waters, Milford, MA, U.S.A.) equipped with a Waters 2487 Dual Absorbance Detector and a Waters 717 plus Autosampler or on a VWR Chromaster (VWR, Radnor, PA, U.S.A.) equipped with a 5160 pump system, a DAD 5430, a 5260 Autosampler, and a MultoHigh100 RP18‐5 μ 250x4 mm column (CS‐Chromatographie Service GmbH, Langerwehe, Germany) using a gradient (H_2_O+0.1 % formic acid/MeOH 80 : 20 isocratic for 5 min to MeOH after additional 45 min and MeOH for additional 10 min) at a flow rate of 1 mL/min or a gradient (H_2_O+0.1 % formic acid/MeOH 60 : 40 isocratic for 5 min to MeOH after additional 25 min and MeOH for additional 10 min) at a flow rate of 1 mL/min with UV‐detection at 245 nm and 280 nm. Only compounds with a purity ≥95 % according to the AUC at UV 245 nm and 280 nm detection were used for biological testing. Compounds **3**, **5**–**11, 13**–**15** and their precursors have been reported previously,^[25]^ **4**, **12**, **16**–**18**, **20** and precursors are shown in the Supporting Information.

**2‐(4‐(3‐Phenylpropoxy)benzamido)isonicotinic acid** (**19**): Ethyl 2‐(4‐(3‐phenylpropoxy)benzamido)isonicotinate (**40**, 35 mg, 0.09 mmol, 1.0 eq) was dissolved in EtOH (5 mL), LiOH (6.4 mg, 0.27 mmol, 3.0 eq) was dissolved in H_2_O (3 mL), both solutions were combined and the mixture was stirred at 50 °C for 12 h. The solvents were then removed in vacuum, the residue was dissolved in H_2_O, the product was precipitated by addition of 2 M HCl, filtered off, washed with cold hexane and dried in vacuum to yield **19** as a colorless solid without further purification (18 mg, 53 %). ^1^H NMR (500 MHz, DMSO) δ=10.84 (s, 1H), 8.70–8.65 (m, 1H), 8.54 (dd, *J*=5.1, 0.6, 1H), 8.04 (d, *J*=8.9, 2H), 7.56 (dd, *J*=5.0, 1.5, 1H), 7.32–7.20 (m, 5H), 7.04 (d, *J*=8.9, 2H), 4.06 (t, *J*=6.3, 2H), 2.78–2.73 (m, 2H), 2.07–2.00 (m, 2H). ^13^C NMR (126 MHz, DMSO) δ=166.19, 165.57, 161.75, 153.37, 148.86, 141.32, 140.04, 131.39, 130.15, 128.39, 125.90, 118.54, 114.27, 114.12, 113.91, 67.03, 33.37, 30.27. MS (ESI‐): *m/z* 375.34 ([M−H]^−^). HRMS (MALDI): *m/z* calc. for C_22_H_20_N_2_O_4_Na 399.13153, found 399.13135 ([M+Na]^+^).

**Methyl 4‐(3‐phenylpropoxy)benzoate** (**29**): Methyl 4‐hydroxybenzoate (**27**, 0.45 g, 2.9 mmol, 1.3 eq), a caesium carbonate (2.3 g, 6.9 mmol, 3.0 eq) and (3‐bromopropyl)benzene (**28**, 0.45 mL, 2.3 mmol, 1.0 eq) were dissolved in DMF (35 mL). The mixture was stirred under reflux for 12 h. After cooling to room temperature, aqueous hydrochloric acid (2 M, 30 mL) was added, phases were separated, and the aqueous layer was extracted with EtOAc (3x 30 mL). The combined organic layers were dried over MgSO_4_, and the solvents were evaporated in vacuum. The crude product was purified by column chromatography using EtOAc/hexane (7 : 3) as mobile phase. **29** was obtained as a yellow oil (0.62 g, 80 %). ^1^H NMR (500 MHz, DMSO) δ=7.90 (d, *J*=8.9, 2H), 7.29–7.26 (m, 2H), 7.24–7.16 (m, 3H), 7.02 (d, *J*=8.9, 2H), 4.02 (t, *J*=6.4, 2H), 3.80 (s, 3H), 2.76–2.70 (m, 2H), 2.05–1.99 (m, 2H). ^13^C NMR (126 MHz, DMSO) δ=165.91, 162.52, 141.25, 131.25, 128.36, 128.34, 125.88, 121.78, 114.42, 67.07, 51.77, 31.39, 30.21. MS (ESI+): *m/z* 293.17 ([M+Na]^+^).

**4‐(3‐Phenylpropoxy)benzoic acid** (**30**): Methyl 4‐(3‐phenylpropoxy)benzoate (**29**, 0.62 g, 2.3 mmol, 1.0 eq) was dissolved in EtOH (30 mL), LiOH (0.10 g, 4.6 mmol, 2.0 eq) was dissolved in H_2_O (10 mL), both solutions were combined and the mixture was stirred at 50 °C for 12 h. The solvents were then removed in vacuum, the residue was dissolved in H_2_O, the product was precipitated by addition of 2 M HCl, filtered off, washed with cold hexane and dried in vacuum to yield **30** as a colorless solid (0.51 g, 86 %) which was used without further purification. ^1^H NMR (500 MHz, DMSO) δ=7.87 (d, *J*=8.9, 2H), 7.30–7.27 (m, 2H), 7.25–7.16 (m, 3H), 7.00 (d, *J*=8.9, 2H), 4.03 (t, *J*=6.4, 2H), 2.77–2.70 (m, 2H), 2.06–2.00 (m, 2H). ^13^C NMR (126 MHz, DMSO) δ=167.04, 162.25, 141.30, 131.40, 128.40, 128.38, 125.91, 122.91, 114.28, 67.03, 31.41, 30.24. MS (ESI‐): *m/z* 255.09 ([M−H]^−^).

**Ethyl 2‐(4‐(3‐phenylpropoxy)benzamido)isonicotinate** (**40**): 4‐(3‐Phenylpropoxy)benzoic acid (**30**, 0.13 g, 0.51 mmol, 1.0 eq) was dissolved in a 3 : 1 mixture of chloroform and DMF (30 mL), EDC⋅HCl (0.15 g, 0.77 mmol, 1.5 eq), 4‐DMAP (0.09 g, 0.77 mmol, 1.5 eq) and ethyl 2‐aminoisonicotinate (**36**, 85 mg, 0.51 mmol, 1.0 eq) were added, and the mixture was stirred at 50 °C for 12 h. After cooling to room temperature, aqueous hydrochloric acid (5 %, 30 mL) was added, phases were separated, and the aqueous layer was extracted with EtOAc (3x30 mL). The combined organic layers were dried over Na_2_SO_4_ and the solvents were evaporated in vacuum. The crude product was purified by column chromatography using EtOAc/hexane (5 : 1) as mobile phase. **40** was obtained as a yellow solid (35 mg, 18 %). ^1^H NMR (500 MHz, CDCl_3_) δ=9.47 (s, 1H), 8.98 (s, 1H), 8.37 (s, 1H), 7.96 (d, *J*=8.3, 2H), 7.65 (s, 1H), 7.31–7.24 (m, 3H), 7.20–7.17 (m, 2H), 6.96 (d, *J*=8.2, 2H), 4.42 (q, *J*=7.1, 2H), 4.02 (t, *J*=6.2, 2H), 2.81 (t, *J*=7.5, 2H), 2.16–2.08 (m, 2H), 1.41 (t, *J*=7.1, 3H). ^13^C NMR (126 MHz, CDCl_3_) δ=165.47, 164.66, 162.75, 152.58, 146.75, 141.33, 141.27, 129.70, 128.58, 128.56, 126.13, 125.57, 119.07, 114.69, 114.64, 67.25, 62.21, 34.01, 21.04, 14.31. MS (ESI+): *m/z* 405.20 ([M+H]^+^).

### In vitro characterization

**LTA4H assay**. Recombinant LTA4H was cloned, expressed and purified as described previously.[^36^, ^37^] In brief, LTA4H was overexpressed in E. coli BL21(DE3)RIPL‐Codon Plus cells (Invitrogen) which were grown in a 1 L culture of Miller's LB Broth Base™ (Invitrogen) at 37 °C and 180 rpm until an OD_600_ of approximately 0.8 before protein expression was induced by the addition of 400 μM IPTG (AppliChem). Temperature was reduced to 21 °C and cultures were harvested after 18 h by centrifugation. Cell pellets were suspended in buffer A (50 mM Tris, 500 mM NaCl, and 20 mM imidazole, HCl, pH 8) supplemented with approximately 0.5 g DNAse (AppliChem) and an EDTA‐free protease inhibitor complete tablet (Roche) before lysis. Cell debris were removed by centrifugation, the supernatant was filtered (0.45 μM syringe filter) before further purification by immobilized metal ion affinity chromatography on a 5 ml HisTrap HP (GE Healthcare) using a step gradient protocol. Buffer A was used as running buffer, while buffer B (identical to buffer A with an imidazole concentration of 400 mM) was used as elution buffer. Protein was eluted at 100 % B. Fractions containing the protein were further purified on a HiLoad 16/600 Superdex 200 pg^TM^ column (GE Healthcare) using buffer C (50 mM Tris, 500 mM NaCl, HCl pH 8). Pure protein in buffer (50 mM Tris, 50 mM NaCl, pH=8) was frozen in liquid nitrogen and stored at −80 °C. A fluorescence‐based LTA4H enzyme activity assay was performed using this recombinant protein according the published protocol^[30]^ by Wittmann et al. with L‐arginine‐7‐amino‐4‐methylcoumarine (Sigma Aldrich) as fluorogenic substrate which is cleaved by LTA4H to L‐arginine and fluorescent 7‐amino‐4‐methylcoumarine. The assay was performed in black polystyrol 96‐well plates in a final volume of 100 μL. Test compound solution in DMSO (1 μL) or DMSO alone as control were incubated with 89 μl protein mixture (containing LTA4H, buffer and Triton‐X‐100). After 30 min, 10 μl of substrate mixture (containing L‐arginin‐7‐amido‐4‐methylcumarine and DMSO in buffer) were added. The final solutions contained test compound (up to 100 μM), LTA4H (0.1 μM), Triton‐X‐100 (0.001 %), Tris (48.7 mM), NaCl (48.7 mM), DMSO (1.4 %), and L‐arginin‐7‐amido‐4‐methylcumarine (182 μM). After substrate addition, fluorescence intensity was measured every minute for a duration of 45 min on a Tecan Infinite F200 Pro Plate Reader (Tecan Deutschland GmbH, Germany) at λ_em_=360 nm and λ_ex_=465 nm at room temperature. A blank measurement containing no protein was performed and treated as 0 % conversion control. All samples were measured in triplicates and each experiment was conducted in three independent repeats. For IC_50_ calculation, the slope of the substrate conversion for every well was determined, mean and standard derivation per triplicate were obtained and normalized using the blank experiment as 0 % conversion and the DMSO control as 100 % conversion in Microsoft Office Excel (2013). The results of three different experiments were analyzed using GraphPad Prism 7 by transforming the concentration to logarithmic values and using the “log(inhibitor) vs. normalized response‐variable slope” fit to determine IC_50_ values. Bestatin (IC_50_ 0.30±0.01 μM) served as reference LTA4H inhibitor.^[30]^

**Full‐length FXR:RXR reporter gene assay**. HeLa cells were grown in DMEM high glucose supplemented with 10 % FCS, sodium pyruvate (1 mM), penicillin (100 U/mL) and streptomycin (100 μg/mL) at 37 °C and 5 % CO_2_ and seeded in 96‐well plates 24 h before transfection with a density of 8000 cells per well. 3.5 h before transfection, medium was changed to DMEM high glucose, supplemented with sodium pyruvate (1 mM), penicillin (100 U/mL), streptomycin (100 μg/mL) and 0.5 % charcoal‐stripped FCS. Cells were then transiently transfected with using the calcium phosphate transfection method with pGL3basic‐BSEP[^26^, ^27^] (containing a shortened construct of the promotor of the bile salt export protein (BSEP) cloned into the SacI/NheI cleavage site in front of the luciferase gene), pcDNA3‐hFXR^[26]^ and pSG5‐hRXR^[38]^ coding for the full‐length human nuclear receptors FXR and RXRα, and pRL‐SV40 (Promega) for normalization of transfection efficiency and cell growth. 16 h after transfection, medium was changed to DMEM high glucose, supplemented with sodium pyruvate (1 mM), penicillin (100 U/mL), streptomycin (100 μg/mL) and 0.5 % charcoal‐stripped FCS. 24 h after transfection, medium was changed to DMEM without phenol red, supplemented with sodium pyruvate (1 mM), penicillin (100 U/mL), streptomycin (100 μg/mL), L‐glutamine (2 mM) and 0.5 % charcoal‐stripped FCS, now additionally containing 0.1 % DMSO and the respective test compound or 0.1 % DMSO alone as untreated control. Each sample was tested in triplicate wells and each experiment was repeated independently at least three times. Following 24 h incubation with the test compounds, cells were assayed for luciferase activity using Dual‐Glo Luciferase Assay System (Promega) according to the manufacturer's protocol. Luminescence was measured with a Tecan Spark M luminometer (Tecan). Normalization of transfection efficiency and cell growth was done by division of firefly luciferase data by Renilla luciferase data multiplied by 1000 resulting in relative light units (RLU). Fold activation was obtained by dividing the mean RLU of the tested compound at a respective concentration by the mean RLU of untreated control. EC_50_ and standard error of the mean values were calculated with the mean fold activation values of at least three independent experiments with SigmaPlot 10.0 (Systat Software GmbH, Erkrath, Germany) using a four‐parameter logistic regression. GW4064 (EC_50_ 0.5±0.2 μM, 3 μM defined as 100 % activation), CDCA (EC_50_ 18±1 μM, 88±3 % eff.) and obeticholic acid (EC_50_ 0.16±0.02 μM, 87±3 % eff.) served as reference FXR agonists.^[27]^

**Hybrid reporter gene assays**. Nuclear receptor modulation was determined in hybrid reporter gene assays using the Gal4‐fusion receptor plasmids pFA‐CMV‐hPPARα‐LBD,^[39]^ pFA‐CMV‐hPPARγLBD,^[39]^ pFA‐CMV‐hPPARδ‐LBD,^[39]^ pFA‐CMV‐hLXRα‐LBD,^[40]^ pFACMV‐hLXRβ‐LBD,^[40]^ pFA‐CMV‐hRXRα‐LBD,^[41]^ pFA‐CMV‐hRARα‐LBD,^[41]^ pFA‐CMV‐hVDR‐LBD^[41]^ and pFA‐CMV‐hCAR‐LBD^[41]^ coding for the hinge region and ligand binding domain (LBD) of the canonical isoform of the respective nuclear receptor, pFR‐Luc (Stratagene) as reporter plasmid and pRL‐SV40 (Promega) for normalization of transfection eﬃciency. HEK293T cells were grown in DMEM high glucose, supplemented with 10 % FCS, sodium pyruvate (1 mM), penicillin (100 U/mL), and streptomycin (100 μg/mL) at 37 °C and 5 % CO_2_, and seeded in 96‐well plates (2.5 ⋅ 10^4^ cells/well) the day before transfection. Before transfection, medium was changed to Opti‐MEM without supplements. Transient transfection was carried out using Lipofectamine LTX reagent (Invitrogen) according to the manufacturer's protocol with pFR‐Luc (Stratagene), pRL‐SV40 (Promega), and the respective hybrid receptor construct pFA‐CMV‐hNR‐LBD. 5 h after transfection, medium was changed to Opti‐MEM supplemented with penicillin (100 U/mL), streptomycin (100 μg/mL), now additionally containing 0.1 % DMSO and the respective test compounds or 0.1 % DMSO alone as untreated control. Each concentration was tested in duplicates, and each experiment was repeated independently at least three times. Following overnight (12–14 h) incubation with the test compounds, cells were assayed for luciferase activity using Dual‐Glo™ luciferase assay system (Promega) according to the manufacturer's protocol. Luminescence was measured with an Infinite M200 luminometer (Tecan Deutschland GmbH, Germany). Normalization of transfection eﬃciency and cell growth was done by division of firefly luciferase data by renilla luciferase data and multiplying the value by 1000 resulting in relative light units (RLU). Fold activation was obtained by dividing the mean RLU of test compounds at a respective concentration by the mean RLU of untreated control. Relative activation was obtained by dividing the fold activation of a test compound at a respective concentration by the fold activation of a respective reference agonist at 1 μM (PPARα, GW7647; PPARγ, pioglitazone; PPARδ, L165,041; LXRα/β, T0901317; RXRα, bexarotene; RARα, tretinoin; VDR, calcitriol; CAR, CITCO). All hybrid assays were validated with the above‐mentioned reference agonists which yielded EC_50_ values in agreement with literature.

## Conflict of interest

The authors declare no conflict of interest.

## Supporting information

As a service to our authors and readers, this journal provides supporting information supplied by the authors. Such materials are peer reviewed and may be re‐organized for online delivery, but are not copy‐edited or typeset. Technical support issues arising from supporting information (other than missing files) should be addressed to the authors.

Supporting Information
