# Supplementary material for: Development and in vitro Profiling of Dual FXR/LTA4H Modulators
Source: ChemMedChem. 2021 May 24;16(15):2366–74. doi: 10.1002/cmdc.202100118 (PMC8453936; doi:10.1002/cmdc.202100118)
Supplement: Supplementary file 1 — Supporting Information [file CMDC-16-2366-s001.pdf]

ChemMedChem

Supporting Information

Development and in vitro Profiling of Dual FXR/LTA4H Modulators

Simone Schierle[+], Steffen Brunst[+], Moritz Helmstädter, Roland Ebert, Jan S. Kramer, Dieter Steinhilber, Ewgenij Proschak,* and Daniel Merk*

Table of Contents

Supplementary Figures …………………………………………………………………. 2
Supplementary Methods ……………………………………………………………... 3
Chemistry ……………………………………………………………………………… 3
In vitro biological evaluation ……………………………………………………… 9
Supplementary References ………………………………………………………….. 11

Supplementary Figures

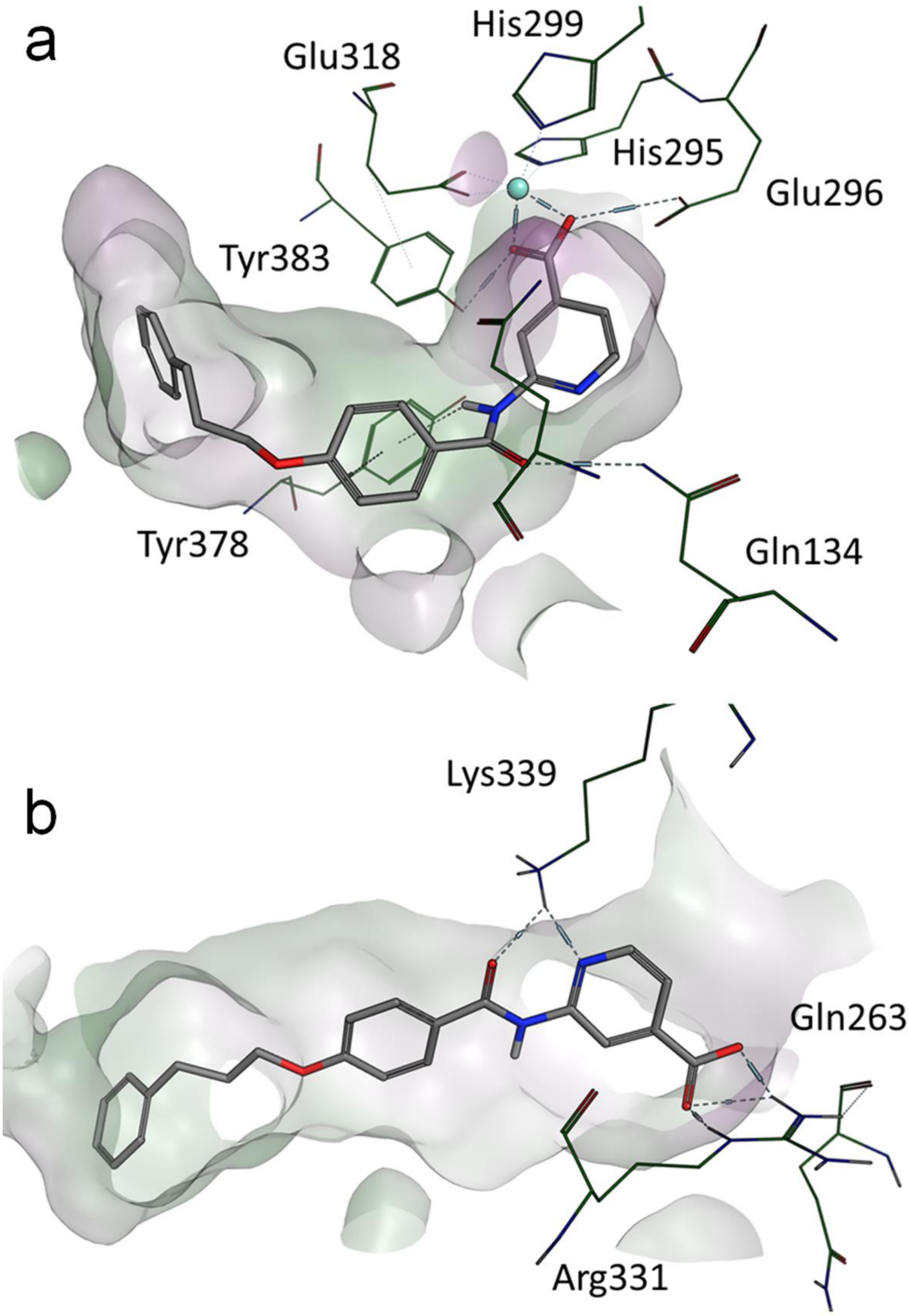

Supplementary Figure 1. Molecular docking of FXRa/LTA4Hi **19** to the LTA4H active site (a, pdb ID 3FHE[1]) and the FXR ligand binding site (b, pdb ID 4QE8[2]).

Supplementary Methods

Chemistry

General. All chemicals and solvents were of reagent grade and used without further purification unless otherwise specified. All reactions were conducted in oven-dried glassware under argon atmosphere and in absolute solvents. NMR spectra were recorded on a Bruker AV 500, Bruker spectrometer (Bruker Corporation, Billerica, MA, USA). Chemical shifts (δ) are reported in ppm relative to tetramethylsilane (TMS) as reference. Multiplicity is reported: s, singlet; d, doublet; dd, doublet of doublets; ddd, doublet of doublet of doublets; t, triplet; m, multiplet. Approximate coupling constants (*J*) are shown in hertz (Hz). Mass spectra were obtained on a VG Platform II (Thermo Fischer Scientific, Inc., Waltham, MA, USA) using electrospray ionization (ESI). High resolution mass spectra were recorded on a MALDI LTQ ORBITRAP XL instrument (Thermo Fisher Scientific). Compound purity was analyzed on a Waters 600 Controller HPLC (Waters, Milford, MA, U.S.A.) equipped with a Waters 2487 Dual Absorbance Detector and a Waters 717 plus Autosampler or on a VWR Chromaster (VWR, Radnor, PA, U.S.A.) equipped with a 5160 pump system, a DAD 5430, a 5260 Autosampler, and a MultoHigh100 RP18-5 µ 250x4 mm column (CS-Chromatographie Service GmbH, Langerwehe, Germany) using a gradient (H_2O+0.1% formic acid/MeOH 80:20 isocratic for 5 min to MeOH after additional 45 min and MeOH for additional 10 min) at a flow rate of 1 mL/min or a gradient (H_2O+0.1% formic acid/MeOH 60:40 isocratic for 5 min to MeOH after additional 25 min and MeOH for additional 10 min) at a flow rate of 1 mL/min with UV-detection at 245 nm and 280 nm. Only compounds with a purity ≥95% according to the AUC at UV 245 nm and 280 nm detection were used for biological testing. Compounds **3**, **5**-**11**, **13**-**15** as well as its precursors have been reported previously[3].

General procedure A for ester hydrolysis with LiOH (**12**,**16**-**17**,**19**-**20**, **30**): The respective ester (**26**, **29** or **38**-**41**, 1 eq) was dissolved in EtOH. LiOH (3-5 eq) was dissolved in H_2O. Subsequently, both reaction mixtures were combined and stirred at 50 °C for 12 h. The solvents were removed, the residue was dissolved in H_2O, the product was precipitated by addition of 2 N HCl, filtered off, washed with cold hexane and dried in vacuum.

General procedure B for amide coupling (**26**, **29**, **32**, **38**-**41**): The respective benzoic acid (**24** or **30**, 1.0 eq) was dissolved in chloroform and DMF (3:1). EDC·HCl (1.5 eq), 4-DMAP (1.5 eq) and respective anilines (**31** or **34**-**37**, 1.0 eq) were added. The reaction mixture was stirred at 50 °C for 12 h. Hydrochloric acid (5%) was then added, phases were separated, and the product was extracted with EtOAc (3x). The combined organic layers were dried over Na_2SO_4 and the solvents were evaporated in vacuum. Further purification was performed by column chromatography using EtOAc/hexane as mobile phase.

3-(4-([1,1'-Biphenyl]-3-yloxy)benzamido)benzoic acid (**12**): Preparation according to general procedure A using ethyl 3-(4-([1,1'-biphenyl]-3-yloxy)benzamido)benzoate (**26**, 0.10 g, 0.23 mmol, 1.0 eq), LiOH (17 mg, 0.69 mmol, 3.0 eq) to yield **12** as a colorless solid without further purification (70 mg, 74%). ^{1}H NMR (500 MHz, DMSO) δ = 8.43 – 8.40 (m, 1H), 8.09 – 7.96 (m, 3H), 7.74 – 7.64 (m, 3H), 7.58 – 7.51 (m, 2H), 7.51 – 7.44 (m, 3H), 7.42 – 7.35 (m, 2H), 7.18 (d, *J*=8.8, 2H), 7.15 – 7.09 (m, 1H). ^{13}C NMR (126 MHz, DMSO) δ = 167.22, 164.88, 159.82, 156.17, 142.46, 139.47, 139.21, 130.83, 130.08, 129.31, 129.02, 128.86, 128.43, 127.93, 127.71, 126.80, 124.36, 122.74, 121.09, 118.44, 117.67, 117.62. MS (ESI+): *m/z* 432.07 ([M+Na]$^{+}$). HRMS (MALDI): *m/z* calc. for $C_{26}H_{19}NO_4Na$ 432.12063, found 432.12033 ([M+Na]$^{+}$).

4-(4-(3-Phenylpropoxy)benzamido)benzoic acid (**16**): Preparation according to general procedure A using ethyl 4-(4-(3-phenylpropoxy)benzamido)benzoate (**38**, 0.16 g, 0.39 mmol, 1.0 eq), LiOH (0.03 g, 1.2 mmol, 3.0 eq) to yield **16** as a colorless solid without further purification (95 mg, 68%). ^{1}H NMR (500 MHz, DMSO) δ = 10.35 (s, 1H), 7.98 – 7.90 (m, 6H), 7.31 – 7.17 (m, 5H), 7.07 (d, *J*=8.8, 2H), 4.06 (t, *J*=6.4, 2H), 2.79 – 2.73 (m, 2H), 2.09 – 2.02 (m, 2H). ^{13}C NMR (126 MHz, DMSO) δ = 166.97, 165.25, 161.51, 143.52, 141.29, 130.17, 129.80, 128.36, 128.35, 126.53, 125.87, 125.22, 119.37, 114.12, 67.00, 31.39, 30.24. MS (ESI+): *m/z* 376.37 ([M+H]$^{+}$). HRMS (MALDI): *m/z* calc. for $C_{23}H_{21}NO_4$ 376.15433, found 376.15468 ([M+H]$^{+}$).

2-(3-(4-(3-Phenylpropoxy)benzamido)phenyl)acetic acid (**17**): Preparation according to general procedure A using methyl 2-(3-(4-(3-phenylpropoxy)benzamido)phenyl)acetate (**39**, 0.15 g, 0.37 mmol, 1.0 eq), LiOH (0.03 g, 1.1 mmol, 3.0 eq) to yield **17** as a colorless solid without further purification (0.11 g, 76%). ^{1}H NMR (500 MHz, DMSO) δ = 10.10 (s, 1H), 7.96 (d, *J*=8.8, 2H), 7.71 – 7.64 (m, 2H), 7.30 – 7.23 (m, 5H), 7.20 – 7.17 (m, 1H), 7.04 (d, *J*=8.8, 2H), 6.97 (d, *J*=7.5, 1H), 4.05 (t, *J*=6.4, 2H), 3.53 (s, 2H), 2.78 – 2.73 (m, 2H), 2.08 – 2.00 (m, 2H). ^{13}C NMR (126 MHz, DMSO) δ = 172.68, 164.85, 161.26, 141.31, 139.32, 135.61, 129.64, 128.37, 126.87, 125.88, 124.50, 121.21, 118.64, 114.03, 66.96, 41.28, 31.42, 30.28. MS (ESI+): *m/z* 412.19 ([M+Na]$^{+}$). HRMS (MALDI): *m/z* calc. for $C_{24}H_{23}NO_4Na$ 412.15193, found 412.15318 ([M+Na]$^{+}$).

***N*-(3-(Methylsulfonamido)phenyl)-4-(3-phenylpropoxy)benzamide** (**18**): *N*-(3-Aminophenyl)-4-(3-phenylpropoxy)benzamide (**33**, 0.15 g, 0.43 mmol, 1.0 eq) was dissolved in 20 mL THF/pyridine (10:1), methanesulfonyl chloride (0.03 mL, 1.3 mmol, 3.0 eq) was added and the reaction mixture was stirred at rt for 6 h. Hydrochloric acid (2 N, 30 mL) was then added, phases were separated, and the product was extracted with ethyl acetate (3x 20 mL). The combined organic layers were dried over Na_2SO_4 and the solvents were evaporated in vacuum. Further purification was performed by column chromatography using EtOAc/hexane (5:1) as mobile phase to yield **18** as a colorless solid (0.11 g, 61%). ^{1}H NMR (500 MHz, DMSO) δ = 10.13 (s, 1H), 9.75 (s, 1H), 7.94 (d, *J*=8.9, 2H), 7.73 (t, *J*=2.0, 1H), 7.54 – 7.50 (m, 1H), 7.32 – 7.22 (m, 5H), 7.22 – 7.17 (m, 1H), 7.05 (d, *J*=8.9, 2H), 6.93 – 6.91 (m, 1H), 4.06 (t, *J*=6.4, 2H), 3.00 (s, 3H), 2.79 – 2.73 (m, 2H), 2.08 – 2.02 (m, 2H). ^{13}C NMR (126 MHz, DMSO)

δ = 164.92, 161.30, 141.29, 140.17, 138.62, 130.03, 129.65, 129.22, 128.35, 126.77, 125.86, 115.82, 114.85, 114.02, 111.64, 66.95, 40.11, 31.39, 30.25. MS (ESI+): *m/z* 425.14 ($[M+H]^+$). HRMS (MALDI): *m/z* calc. for $C_{23}H_{24}N_2O_4SNa$ 447.13490, found 447.13452 ($[M+Na]^+$).

2-(4-(3-Phenylpropoxy)benzamido)isonicotinic acid (**19**): Preparation according to general procedure A using ethyl 2-(4-(3-phenylpropoxy)benzamido)isonicotinate (**40**, 35 mg, 0.09 mmol, 1.0 eq), LiOH (6.4 mg, 0.27 mmol, 3.0 eq) to yield **19** as a colorless solid without further purification (18 mg, 53%). ^1H NMR (500 MHz, DMSO) δ = 10.84 (s, 1H), 8.70 – 8.65 (m, 1H), 8.54 (dd, *J*=5.1, 0.6, 1H), 8.04 (d, *J*=8.9, 2H), 7.56 (dd, *J*=5.0, 1.5, 1H), 7.32 – 7.20 (m, 5H), 7.04 (d, *J*=8.9, 2H), 4.06 (t, *J*=6.3, 2H), 2.78 – 2.73 (m, 2H), 2.07 – 2.00 (m, 2H). ^{13}C NMR (126 MHz, DMSO) δ = 166.19, 165.57, 161.75, 153.37, 148.86, 141.32, 140.04, 131.39, 130.15, 128.39, 125.90, 118.54, 114.27, 114.12, 113.91, 67.03, 33.37, 30.27. MS (ESI-): *m/z* 375.34 ($[M-H]^-$). HRMS (MALDI): *m/z* calc. for $C_{22}H_{20}N_2O_4Na$ 399.13153, found 399.13135 ($[M+Na]^+$).

2-(4-(3-Phenylpropoxy)benzamido)oxazole-4-carboxylic acid (**20**): Preparation according to general procedure A using ethyl 4-(4-(3-phenylpropoxy)benzamido)oxazole-2-carboxylate (**41**, 55 mg, 0.14 mmol, 1.0 eq), LiOH (0.01 g, 0.42 mmol, 3.0 eq) to yield **20** as a yellow solid without further purification (14 mg, 27%).^1H NMR (500 MHz, DMSO) δ = 7.77 (d, *J*=8.0, 1H), 7.45 – 7.39 (m, 2H), 7.29 – 7.23 (m, 2H), 7.22 – 7.15 (m, 3H), 6.93 – 6.86 (m, 2H), 3.98 (t, *J*=6.3, 2H), 2.74 – 2.69 (m, 2H), 2.03 – 1.95 (m, 2H). ^{13}C NMR (126 MHz, DMSO) δ = 167.82, 167.78, 166.63, 160.10, 154.27, 141.27, 129.60, 128.97, 128.62, 128.33, 128.30, 125.84, 113.69, 66.73, 31.33, 30.16. MS (ESI+): *m/z* 367.20 ($[M+H]^+$). HRMS (MALDI): *m/z* calc. for $C_{20}H_{18}N_2O_5Na$ 389.11079, found 389.11083 ($[M+Na]^+$).

Ethyl 4-([1,1'-biphenyl]-3-yloxy)benzoate (**23**): 3-hydroxydiphenyl (**21**, 0.17 g, 1.0 mmol, 1.0 eq) and (4-(ethoxycarbonyl)phenyl)boronic acid (**22**, 0.19 g, 1.0 mmol, 1.0 eq) were dissolved in DMF (40 mL), then pyridine (0.40 mL, 5.0 mmol, 5.0 eq) and $Cu(OAc)_2$ (0.18 g, 1.0 mmol, 1.0 eq) were added. The mixture was stirred under reflux for 12 h. After cooling to room temperature, aqueous hydrochloric acid (2 M, 30 mL) was added, phases were separated, and the aqueous layer was extracted with EtOAc (3x 30 mL). The combined organic layers were dried over $MgSO_4$, and the solvents were evaporated in vacuum. The crude product was purified by column chromatography using EtOAc/hexane (3:1) as mobile phase. **23** was obtained as a yellow oil (0.21 g, 66%). ^1H NMR (500 MHz, DMSO) δ = 7.98 (d, *J*=8.9, 2H), 7.70 – 7.64 (m, 2H), 7.61 – 7.34 (m, 7H), 7.12 (d, *J*=8.9, 2H), 4.29 (q, *J*=7.1, 2H), 1.31 (t, *J*=7.1, 3H). ^{13}C NMR (126 MHz, DMSO) δ = 165.17, 161.19, 155.62, 142.51, 139.10, 131.57, 130.89, 129.01, 127.94, 126.81, 126.59, 123.08, 118.86, 118.09, 117.47, 60.58, 14.21. MS (ESI+): *m/z* 341.07 ($[M+Na]^+$).

4-([1,1'-Biphenyl]-3-yloxy)benzoic acid (**24**): Preparation according to general procedure A using ethyl 4-([1,1'-biphenyl]-3-yloxy)benzoate (**23**, 0.21 g, 0.66 mmol, 1.0 eq), LiOH (79 mg, 3.3 mmol, 5.0 eq) to yield **24** as a colorless solid (0.18 g, 95%). ^1H NMR (500 MHz, DMSO) δ = 7.96 (d, *J*=8.8, 2H), 7.70 – 7.66 (m, 2H), 7.56 – 7.53 (m, 2H), 7.48 – 7.45 (m, 2H), 7.42 –

7.36 (m, 2H), 7.13 – 7.08 (m, 3H). ^{13}C NMR (126 MHz, DMSO) δ = 167.22, 161.42, 156.18, 142.96, 139.59, 132.19, 131.34, 129.48, 128.40, 127.28, 125.75, 123.48, 119.32, 118.56, 117.79. MS (ESI-): *m/z* 288.96 ([M-H]$^-$).

Ethyl 3-(4-([1,1'-biphenyl]-3-yloxy)benzamido)benzoate (**26**): Preparation according to general procedure B using 4-([1,1'-biphenyl]-3-yloxy)benzoic acid (**24**, 0.18 g, 0.65 mmol, 1.0 eq), EDC·HCl (0.19 g, 0.98 mmol, 1.5 eq), 4-DMAP (0.12 g, 0.98 mmol, 1.5 eq) and ethyl 3-aminobenzoate (**25**, 0.11 g, 0.65 mmol, 1.0 eq). The crude product was purified by column chromatography using EtOAc/hexane (3:1) as mobile phase. **26** was yielded as a colorless solid (0.1g, 36%). ^{1}H NMR (500 MHz, DMSO) δ = 8.43 (t, *J*=1.8, 1H), 8.10 – 8.02 (m, 4H), 7.70 – 7.67 (m, 3H), 7.55 – 7.44 (m, 6H), 7.18 (d, *J*=8.8, 2H), 7.13 – 7.09 (m, 1H), 4.33 (q, *J*=7.1, 2H), 1.33 (t, *J*=7.2, 3H). ^{13}C NMR (126 MHz, DMSO) δ = 165.65, 164.94, 159.87, 156.15, 142.46, 139.63, 139.09, 132.45, 130.84, 130.12, 129.07, 129.03, 127.94, 126.84, 126.80, 126.78, 124.71, 122.76, 120.78, 118.47, 117.69, 117.61, 60.82, 14.21. MS (ESI+): no molecular ion.

Methyl 4-(3-phenylpropoxy)benzoate (**29**): Methyl 4-hydroxybenzoate (**27**, 0.45 g, 2.9 mmol, 1.3 eq), cesium carbonate (2.3 g, 6.9 mmol, 3.0 eq) and (3-bromopropyl)benzene (**28**, 0.45 mL, 2.3 mmol, 1.0 eq) were dissolved in DMF (35 mL). The mixture was stirred under reflux for 12 h. After cooling to room temperature, aqueous hydrochloric acid (2 M, 30 mL) was added, phases were separated, and the aqueous layer was extracted with EtOAc (3x 30 mL). The combined organic layers were dried over $MgSO_4$, and the solvents were evaporated in vacuum. The crude product was purified by column chromatography using EtOAc/hexane (7:3) as mobile phase. **29** was obtained as a yellow oil (0.62 g, 80%). ^{1}H NMR (500 MHz, DMSO) δ = 7.90 (d, *J*=8.9, 2H), 7.29 – 7.26 (m, 2H), 7.24 – 7.16 (m, 3H), 7.02 (d, *J*=8.9, 2H), 4.02 (t, *J*=6.4, 2H), 3.80 (s, 3H), 2.76 – 2.70 (m, 2H), 2.05 – 1.99 (m, 2H). ^{13}C NMR (126 MHz, DMSO) δ = 165.91, 162.52, 141.25, 131.25, 128.36, 128.34, 125.88, 121.78, 114.42, 67.07, 51.77, 31.39, 30.21. MS (ESI+): *m/z* 293.17 ([M+Na]$^+$).

4-(3-Phenylpropoxy)benzoic acid (**30**): Preparation according to general procedure A using methyl 4-(3-phenylpropoxy)benzoate (**29**, 0.62 g, 2.3 mmol, 1.0 eq), LiOH (0.10 g, 4.6 mmol, 2.0 eq) to yield **30** as a colorless solid. **30** was used without further purification (0.51 g, 86%). ^{1}H NMR (500 MHz, DMSO) δ = 7.87 (d, *J*=8.9, 2H), 7.30 – 7.27 (m, 2H), 7.25 – 7.16 (m, 3H), 7.00 (d, *J*=8.9, 2H), 4.03 (t, *J*=6.4, 2H), 2.77 – 2.70 (m, 2H), 2.06 – 2.00 (m, 2H). ^{13}C NMR (126 MHz, DMSO) δ = 167.04, 162.25, 141.30, 131.40, 128.40, 128.38, 125.91, 122.91, 114.28, 67.03, 31.41, 30.24. MS (ESI-): *m/z* 255.09 ([M-H]$^-$).

***N*-(3-Nitrophenyl)-4-(3-phenylpropoxy)benzamide** (**32**): Preparation according to general procedure B using 4-(3-phenylpropoxy)benzoic acid (**30**, 0.41 g, 1.6 mmol, 1.0 eq), EDC·HCl (0.46 g, 2.4 mmol, 1.5 eq), 4-DMAP (0.33 g, 2.4 mmol, 1.5 eq) and 3-nitroaniline (**31**, 0.22 g, 1.6 mmol, 1.0 eq). The crude product was purified by column chromatography using EtOAc/hexane (5:1) as mobile phase. **32** was yielded as a yellow solid (0.48 g, 80%). ^{1}H NMR (500 MHz, DMSO) δ = 10.52 (s, 1H), 8.80 (t, *J*=2.2, 1H), 8.21 – 8.17 (m, 1H), 7.99 (d, *J*=8.9,

2H), 7.94 (dd, *J*=8.2, 1.5, 1H), 7.64 (t, *J*=8.2, 1H), 7.32 – 7.27 (m, 2H), 7.25 – 7.23 (m, 2H), 7.20 – 7.18 (m, 1H), 7.09 (d, *J*=8.8, 2H), 4.07 (t, *J*=6.3, 2H), 2.79 – 2.73 (m, 2H), 2.09 – 2.02 (m, 2H). ^{13}C NMR (126 MHz, DMSO) δ = 165.34, 161.65, 147.89, 141.27, 140.59, 129.98, 129.79, 128.35, 128.34, 126.09, 126.07, 119.93, 117.82, 114.23, 114.18, 67.02, 31.39, 30.24. MS (ESI+): *m/z* 377.11 ([M+H]$^+$).

***N*-(3-Aminophenyl)-4-(3-phenylpropoxy)benzamide** (**33**): *N*-(3-Nitrophenyl)-4-(3-phenylpropoxy)benzamide (**32**, 0.48 g, 1.3 mmol, 1.0 eq) was dissolved in EtOAc (30 mL) and Pd(C) (loading 10% w/w, 0.14 g, 0.13 mmol, 0.10 eq) was added. The suspension was stirred at room temperature under H_2 atmosphere for 6 h. The mixture was then filtered through celite, the filtrate was dried over $MgSO_4$, and the solvent was evaporated in vacuum to obtain the title compound as purple solid (0.39 g, 88%). ^{1}H NMR (500 MHz, DMSO) δ = 9.76 (s, 1H), 7.91 (d, *J*=8.8, 2H), 7.33 – 7.27 (m, 2H), 7.25 – 7.23 (m, 2H), 7.22 – 7.16 (m, 1H), 7.09 (t, *J*=2.0, 1H), 7.05 – 6.99 (m, 2H), 6.94 (t, *J*=7.9, 1H), 6.88 – 6.82 (m, 1H), 6.29 (ddd, *J*=7.9, 2.1, 0.9, 1H), 5.04 (s, 2H), 4.05 (t, *J*=6.4, 2H), 2.79 – 2.73 (m, 2H), 2.07 – 2.02 (m, 2H). ^{13}C NMR (126 MHz, DMSO) δ = 164.60, 161.06, 148.85, 141.29, 139.89, 129.50, 129.08, 128.69, 128.34, 127.27, 125.86, 113.94, 109.53, 108.37, 103.03, 66.91, 31.40, 30.26. MS (ESI+): *m/z* 347.17 ([M+H]$^+$).

Ethyl 4-(4-(3-phenylpropoxy)benzamido)benzoate (**38**): Preparation according to general procedure B using 4-(3-phenylpropoxy)benzoic acid (**30**, 0.13 g, 0.51 mmol, 1.0 eq), EDC·HCl (0.15 g, 0.77 mmol, 1.5 eq), 4-DMAP (0.09 g, 0.77 mmol, 1.5 eq) and ethyl 4-aminobenzoate (**34**, 84 mg, 0.51 mmol, 1.0 eq) . The crude product was purified by column chromatography using EtOAc/hexane (5:1) as mobile phase. **38** was yielded as a yellow solid (0.16 g, 80%). ^{1}H NMR (500 MHz, DMSO) δ = 10.38 (s, 1H), 7.97 – 7.94 (m, 6H), 7.31 – 7.28 (m, 2H), 7.25 – 7.23 (m, 2H), 7.21 – 7.17 (m, 1H), 7.09 – 7.05 (m, 2H), 4.29 (q, *J=7.1*, 2H), 4.06 (t, *J=6.4*, 2H), 2.78 – 2.74 (m, 2H), 2.08 – 2.02 (m, 2H), 1.32 (t, *J=7.1*, 3H). ^{13}C NMR (126 MHz, DMSO) δ = 165.42, 165.32, 161.57, 143.86, 141.31, 130.04, 129.85, 128.39, 128.38, 126.47, 125.91, 124.33, 119.50, 114.16, 67.03, 60.46, 31.42, 30.27, 14.25. MS (ESI+): *m/z* 404.24 ([M+H]$^+$).

Methyl 2-(3-(4-(3-phenylpropoxy)benzamido)phenyl)acetate (**39**)**:** Preparation according to general procedure B using 4-(3-phenylpropoxy)benzoic acid (**30**, 0.13 g, 0.51 mmol, 1.0 eq), EDC·HCl (0.15 g, 0.77 mmol, 1.5 eq), 4-DMAP (0.09 g, 0.77 mmol, 1.5 eq) and methyl 2-(3-aminophenyl)acetate (**35**, 84 mg, 0.51 mmol, 1.0 eq) . The crude product was purified by column chromatography using EtOAc/hexane (5:1) as mobile phase. **39** was yielded as a yellow solid (0.15 g, 75%). ^{1}H NMR (500 MHz, $CDCl_3$) δ = 7.82 (d, *J*=8.8, 2H), 7.76 (s, 1H), 7.58 – 7.56 (m, 2H), 7.34 – 7.27 (m, 3H), 7.22 – 7.20 (m, 2H), 7.05 (d, *J*=7.7, 1H), 6.95 (d, *J*=8.8, 2H), 4.02 (t, *J*=6.3, 2H), 3.70 (s, 3H), 3.64 (s, 2H), 2.83 (t, *J*=7.3, 2H), 2.17 – 2.11 (m, 2H). ^{13}C NMR (126 MHz, $CDCl_3$) δ = 172.04, 165.33, 162.16, 141.37, 138.51, 135.06, 129.43, 129.01, 128.65, 128.63, 127.06, 126.20, 125.37, 121.06, 119.08, 114.65, 67.25, 52.26, 41.24, 32.20, 30.79. MS (ESI+): no molecular ion.

Ethyl 2-(4-(3-phenylpropoxy)benzamido)isonicotinate (**40**): Preparation according to general procedure B using 4-(3-phenylpropoxy)benzoic acid (**30**, 0.13 g, 0.51 mmol, 1.0 eq),

EDC·HCl (0.15 g, 0.77 mmol, 1.5 eq), 4-DMAP (0.09 g, 0.77 mmol, 1.5 eq) and ethyl 2-aminoisonicotinate (**36**, 85 mg, 0.51 mmol, 1.0 eq). The crude product was purified by column chromatography using EtOAc/hexane (5:1) as mobile phase. **40** was yielded as a yellow solid (35 mg, 18%). ^{1}H NMR (500 MHz, $CDCl_3$) δ = 9.47 (s, 1H), 8.98 (s, 1H), 8.37 (s, 1H), 7.96 (d, *J*=8.3, 2H), 7.65 (s, 1H), 7.31 – 7.24 (m, 3H), 7.20 – 7.17 (m, 2H), 6.96 (d, *J*=8.2, 2H), 4.42 (q, *J*=7.1, 2H), 4.02 (t, *J*=6.2, 2H), 2.81 (t, *J*=7.5, 2H), 2.16 – 2.08 (m, 2H), 1.41 (t, *J*=7.1, 3H). ^{13}C NMR (126 MHz, $CDCl_3$) δ = 165.47, 164.66, 162.75, 152.58, 146.75, 141.33, 141.27, 129.70, 128.58, 128.56, 126.13, 125.57, 119.07, 114.69, 114.64, 67.25, 62.21, 34.01, 21.04, 14.31. MS (ESI+): *m/z* 405.20 ($[M+H]^+$).

Ethyl 4-(4-(3-phenylpropoxy)benzamido)oxazole-2-carboxylate (**41**): Preparation according to general procedure B using 4-(3-phenylpropoxy)benzoic acid (**30**, 0.13 g, 0.51 mmol, 1.0 eq), EDC·HCl (0.15 g, 0.77 mmol, 1.5 eq), 4-DMAP (0.09 g, 0.77 mmol, 1.5 eq) and ethyl 4-aminooxazole-2-carboxylate (**37**, 80 mg, 0.51 mmol, 1.0 eq). The crude product was purified by column chromatography using EtOAc/hexane (5:1) as mobile phase. **41** was yielded as a yellow solid (55 mg, 28%). ^{1}H NMR (500 MHz, CDCl3) δ = 8.08 (d, J=9.0, 2H), 7.33 – 7.27 (m, 2H), 7.24 – 7.18 (m, 4H), 6.96 (d, J=8.9, 2H), 4.31 (q, J=7.1, 2H), 4.05 (t, J=6.3, 2H), 2.85 – 2.81 (m, 2H), 2.19 – 2.12 (m, 2H), 1.34 (t, J=7.1, 3H). ^{13}C NMR (126 MHz, $CDCl_3$) δ = 167.33, 164.20, 162.48, 141.24, 134.60, 132.98, 129.89, 129.14, 128.64, 128.64, 126.23, 121.31, 114.72, 67.39, 62.15, 32.16, 29.84, 14.34. MS (ESI+): no molecular ion.

In vitro biological evaluation

Aqueous solubility. Aqueous solubility of compounds **3** and **19** were determined using Whatman Uniprep filters (Whatman plc, Maidstone, UK). 5 mg of each compound and 3 mL H_2O dest. were inserted into the Uniprep vessel and the mixture was shaken at 37 °C for 24 h. The mixture was then pressed through the Uniprep filter and the concentration of dissolved compound in filtrate was quantified by HPLC (Varian ProStar, SpectraLab Scientific Inc. equipped with a MultoHigh100 Phenyl 5 µ 240+4 mm column, CS-Chromatographie Service GmbH) using external calibration.

WST-1 assay. WST-1 assay (Roche Diagnostics International AG, Rotkreuz, Schweiz) was performed according to manufacturer's protocol. In brief, HepG2 cells were seeded in DMEM high glucose, supplemented with sodium pyruvate (1 mM), penicillin (100 U/mL), streptomycin (100 µg/mL) and 10% FCS in 96-well plates ($3 \cdot 10^4$ cells/well). After 24 h, medium was changed to DMEM high glucose, supplemented with penicillin (100 U/mL), streptomycin (100 µg/mL) and 1% charcoal stripped FCS and cells were incubated with **3** or **19** (final concentrations 0.1 µM, 1 µM, 10 µM, 30 µM and 100 µM), Revlotron as positive control, and DMEM/1% DMSO as negative control. After 48 h, WST-1 reagent (Roche Diagnostics International AG) was added to each well according to manufacturer's instructions. After 45 min incubation, absorption (450 nm/ reference: 620 nm) was determined with a Tecan Infinite M200 (Tecan Deutschland GmbH, Germany). Each experiment was repeated at least three times in duplicates. Results (expressed as mean percent of untreated control±SEM; n=4; DMSO=100%).

sEH activity assay. Human sEH full length protein was purified as described by Lukin et al.[4]. In brief, the protein was expressed in E.coli BL21 (DE3) and purified on an ÄKTA purifier (GE Healthcare) with a 5 ml HisTrap HP (GE Healthcare) followed by a dialysis with a volume ratio of ~ 1:100 for at least 10 h at 4°C. Pure protein in buffer (50 mM Tris, 500 mM NaCl, 5% glycerol, and pH 8 supplemented with an additional 20% glycerol) was frozen in liquid nitrogen and stored at -80°C. The sEH-hydrolase activity assay procedure was adapted from Hahn et al. and Lukin et al.[4] as described by Brunst et al.[5]. Final compound concentrations were measured between 0 – 300 µM.

Isothermal titration calorimetry. Recombinant FXR LBD for ITC experiments was obtained as described previously[2,5].In brief, the protein was expressed in E.coli BL21 (DE3) and purified on an ÄKTA purifier (GE Healthcare) with a 5 ml HisTrap HP (GE Healthcare). Cleavage of the His_6-tag was achieved by adding self-produced TEV protease in a molecular ratio of 1:10. This was followed by another purification on an ÄKTA purifier with a 5 ml HisTrap HP (GE Healthcare). This time the flow trough was collected and a size-exclusion chromatography (HiLoad 16/600 Superdex 200 pg™, GE Healthcare) was performed. Pure protein in buffer (10 mM Tris, 100 mM NaCl, 5 mM DTT, pH=8.3) was frozen in liquid nitrogen and stored at -80°C. LTA4H protein was obtained as described by Hiesinger et al.[6]. In brief, the protein was expressed in E.coli BL21 (DE3) and purified on an ÄKTA purifier (GE Healthcare) with a 5 ml

HisTrap HP (GE Healthcare) followed by size-exclusion chromatography (HiLoad 16/600 Superdex 200 pg™, GE Healthcare). Pure protein in buffer (50 mM Tris, 50 mM NaCl, pH=8) was frozen in liquid nitrogen and stored at -80°C.

For ITC measurements, the respective protein (FXR or LTA4H) was dialyzed for 4 to 12 hours with the respective buffer in a 1:250 volume ratio, centrifuged at 4°C with 21130 xg for 10 min and diluted to the desired concentration using the dialysis buffer and DMSO to a final concentration of 1% DMSO. Test compounds were prepared by diluting stock solution in dialysis buffer and adding the respective amount of DMSO for a final concentration of 1%. The respective protein (150 µM FXR LBD or 40 µM LTA4H) was titrated to compound (10-60 µM **8** or 15-60 µM **19**) with varying test compound concentrations. ITC measurements were performed on an Affinity ITC (TA-Instruments) at a temperature of 25 °C and a stirring rate of 75 rpm. Control experiments were performed by titrating protein to buffer or buffer to compound with otherwise identical conditions. Each protein-ligand interaction was tested twice with varying ligand concentration. Buffers were the following. FXR LBD: 10 mM Tris, 100 mM NaCl, 5 mM DTT, pH=8.3. LTA4H: 50 mM Tris, 50 mM NaCl, pH=8.0.

Computational Methods

Molecular docking. For molecular docking, MOE2019.0102 (Chemical Computing Group, Montreal, Canada) was used. X-ray structures in complex with compounds displaying structural similarity to compound **19** (FXR: 4QE8[2]; LTA4H: 3FHE[1]) were downloaded from the Protein Data Bank and prepared for docking using the QuickPrep routine with default settings. Ligand **19** was loaded in an MOE database and prepared for docking using Wash routine (default settings, adjusting dominant protonation state at pH 7.4, rebuilding 3D coordinates). Molecular docking was conducted using Similarity placement and induced fit method. Rescoring was performed using GBVI/WSA dG scoring function for generating 5 diverse conformations, followed by energy minimization of the binding site (ligand and residues within 4.5A). The most probable binding mode was selected manually, with special attention to interactions of polar groups.

Supplementary References

(1) Davies, D. R.; Mamat, B.; Magnusson, O. T.; Christensen, J.; Haraldsson, M. H.; Mishra, R.; Pease, B.; Hansen, E.; Singh, J.; Zembower, D.; Kim, H.; Kiselyov, A. S.; Burgin, A. B.; Gurney, M. E.; Stewart, L. J. Discovery of Leukotriene A4 Hydrolase Inhibitors Using Metabolomics Biased Fragment Crystallography. *J. Med. Chem.* **2009**, *52* (15), 4694–4715. https://doi.org/10.1021/jm900259h.

(2) Merk, D.; Sreeramulu, S.; Kudlinzki, D.; Saxena, K.; Linhard, V.; Gande, S. L.; Hiller, F.; Lamers, C.; Nilsson, E.; Aagaard, A.; Wissler, L.; Dekker, N.; Bamberg, K.; Schubert-Zsilavecz, M.; Schwalbe, H. Molecular Tuning of Farnesoid X Receptor Partial Agonism. *Nat. Commun.* **2019**, *10* (1), 2915. https://doi.org/10.1038/s41467-019-10853-2.

(3) Schierle, S.; Schmidt, J.; Kaiser, A.; Merk, D. Selective Optimization of Pranlukast to Farnesoid X Receptor Modulators. *ChemMedChem* **2018**, *13* (23), 2530–2545. https://doi.org/10.1002/cmdc.201800549.

(4) Lukin, A.; Kramer, J.; Hartmann, M.; Weizel, L.; Hernandez-Olmos, V.; Falahati, K.; Burghardt, I.; Kalinchenkova, N.; Bagnyukova, D.; Zhurilo, N.; Rautio, J.; Forsberg, M.; Ihalainen, J.; Auriola, S.; Leppänen, J.; Konstantinov, I.; Pogoryelov, D.; Proschak, E.; Dar’in, D.; Krasavin, M. Discovery of Polar Spirocyclic Orally Bioavailable Urea Inhibitors of Soluble Epoxide Hydrolase. *Bioorg. Chem.* **2018**, *80*, 655–667. https://doi.org/10.1016/j.bioorg.2018.07.014.

(5) Brunst, S.; Kramer, J. S.; Kilu, W.; Heering, J.; Pollinger, J.; Hiesinger, K.; George, S.; Steinhilber, D.; Merk, D.; Proschak, E. Systematic Assessment of Fragment Identification for Multi-target Drug Design. *ChemMedChem* **2020**. https://doi.org/10.1002/cmdc.202000858.

(6) Hiesinger, K.; Schott, A.; Kramer, J. S.; Blöcher, R.; Witt, F.; Wittmann, S. K.; Steinhilber, D.; Pogoryelov, D.; Gerstmeier, J.; Werz, O.; Proschak, E. Design of Dual Inhibitors of Soluble Epoxide Hydrolase and LTA4 Hydrolase. *ACS Med. Chem. Lett.* **2020**, *11* (3), 298–302. https://doi.org/10.1021/acsmedchemlett.9b00330.
